# Evidence-Guided Multimodal Risk Prediction Framework for Severe COVID-19 Outcomes Using EHR and CT Imaging for COVID-19 Clinical Decision Support

**DOI:** 10.3390/bioengineering13080929

**Published:** 2026-08-17

**Authors:** Muhammad Zohaib Khan, Shaukat Wasi, Muhammad Shoaib Siddiqui, Ghufran Ahmed, Muhammad Hussain Mughal, Mohsin Iftikhar

**Affiliations:** 1 Department of Computer Science, Muhammad Ali Jinnah University, Karachi 75400, Pakistan; muhammad.zohaib@jinnah.edu (M.Z.K.); shaukat.wasi@jinnah.edu (S.W.); 2 Faculty of Computer and Information Systems, Islamic University of Madinah, Madinah 42351, Saudi Arabia; 3 Department of Computer Science, School of Computing, National University of Computer and Emerging Sciences (FAST-NUCES), Karachi 75030, Pakistan; ghufran.ahmed@nu.edu.pk; 4 Department of Computer Science, Sukkur IBA University, Sukkur 65020, Pakistan; muhammad.hussain@iba-suk.edu.pk; 5 Faculty of Computer and Information Science, Higher Colleges of Technology, Fujairah P.O. Box 25026, United Arab Emirates; miftikhar@hct.ac.ae

**Keywords:** COVID-19, multimodal risk prediction framework for severe COVID-19 outcomes, multimodal learning, clinical decision support, electronic health records, computed tomography, weighted late fusion, deep learning, ResNet50, gradient boosting

## Abstract

Early identification of COVID-19 patients requiring intensive care is critical for improving treatment prioritization, supporting clinical decision-making, and managing limited hospital resources. While structured electronic health record (EHR) data provide important physiological information, chest computed tomography (CT) imaging contains additional indicators related to disease severity. This study presents a multimodal clinical decision support framework for a multimodal risk prediction framework for severe COVID-19 outcomes using structured emergency clinical features and patient-level CT imaging from the COVID Data for Shared Learning (CDSL) dataset. After multimodal cohort construction, 784 patients with both structured clinical records and CT imaging were included in the analysis. Three predictive settings were evaluated: EHR-only prediction using Gradient Boosting, CT-only prediction using ResNet50-based feature extraction with Logistic Regression, and multimodal prediction using weighted late fusion. The experimental results indicate that the CT-based model surpassed the clinical baseline, yielding an F1-score of 0.42 and an ROC-AUC of 0.772, whereas the EHR-only model achieved scores of 0.30 and 0.715, respectively. Overall, the multimodal fusion framework achieved the strongest results among the approaches tested, reaching an F1-score of 0.47 and an ROC-AUC of 0.782. Taken together, these findings indicate that, although CT imaging alone carries meaningful predictive power for evaluating ICU risk, combining it with clinical data leads to predictions that are more reliable and robust. The proposed framework offers a practical and interpretable foundation for multimodal clinical decision support and demonstrates the potential of combining structured clinical data with medical imaging for intelligent critical care applications.

## 1. Introduction

Recent studies have extensively investigated artificial intelligence-driven COVID-19 risk stratification using electronic health records, laboratory biomarkers, clinical trajectories, and medical imaging modalities, demonstrating the potential of machine learning approaches for multimodal risk prediction framework for severe COVID-19 outcomes, disease severity assessment, and clinical decision support [1,2,3,4,5,6,7,8,9,10]. The outbreak of Coronavirus Disease (COVID-19) put immense strain on healthcare infrastructures across the globe, especially at the intensive care unit level. Timely identification of critically ill individuals was imperative to save lives and rationalize the use of resources [11,12]. Early prediction of intensive care unit (ICU) admission will help medical professionals plan the allocation of resources and optimize outcomes [11]. Late identification may lead to untimely treatment and a higher death rate.

Conventional methods used to predict ICU risk usually depend on structured electronic health records (EHRs). These data include demographics, vital signs, laboratory results, and physiological data. Machine learning approaches, such as Gradient Boosting, Random Forest, and Logistic Regression, have been effective in predicting critical patients based on structured variables [13,14]. Nonetheless, the structured approach alone cannot provide a complete view of lung involvement in COVID-19 patients due to the progressive nature of the disease and its manifestation in medical images [12,15].

Chronic computed tomography (CT) imaging is instrumental in estimating the extent of infection and inflammation in the lungs. Deep learning techniques have been found to possess high discriminative capacity in extracting image features to evaluate patient conditions and predict outcomes [12,15]. Numerous studies showed that CT models outperformed structured clinical models in predicting severe progression and ICU transfer in COVID-19 patients [12,16]. However, using images alone might overlook the crucial physiologic and biochemical information contained in structured medical records.

A combination of structured EHR and imaging data has been found to enhance the reliability of prediction by exploiting complementary information from both sources [17,18]. Late fusion strategies have been particularly effective because they allow independent optimization of each modality before combining predictions at the probability level [17,19]. Weighted late fusion methods further improve interpretability by explicitly controlling the contribution of each modality using a fusion parameter [20,21]. Such approaches are highly suitable for clinical deployment because they preserve model transparency and reduce the risk of unstable feature-level fusion.

Although several multimodal frameworks have been proposed for ICU prediction and COVID-19 severity estimation, most existing work focuses on a multimodal risk prediction framework for severe COVID-19 outcomes, text-based multimodal fusion, or external benchmark datasets such as MIMIC and RadFusion rather than patient-level CT-EHR fusion using real-world COVID cohorts [17,18,20]. Limited work has investigated whether multimodal fusion genuinely improves ICU admission prediction beyond strong CT-only baselines, especially using patient-level representative CT imaging and structured emergency department features.

Recent artificial intelligence-based approaches have demonstrated promising performance for COVID-19 prognosis using either structured clinical information or medical imaging independently. However, these approaches often analyze isolated sources of evidence and may not fully represent the complex decision-making process followed by clinicians, where physiological deterioration patterns and radiological findings are considered jointly.

Although multimodal approaches have emerged, several limitations remain. First, many existing studies focus primarily on diagnostic classification rather than patient-level outcome prediction. Second, longitudinal changes in clinical parameters are frequently simplified into static representations, limiting the ability to capture disease progression. Third, combining heterogeneous modalities remains challenging due to differences in data characteristics and representation spaces. Furthermore, limited attention has been given to developing clinically interpretable fusion strategies that allow understanding of how different evidence sources contribute to the final prediction.

To address these limitations, this study proposes an evidence-guided multimodal framework that integrates structured EHR information and CT imaging representations for COVID-19 clinical outcome prediction. Instead of replacing individual clinical evidence sources, the proposed approach combines complementary information from physiological measurements and radiological characteristics to generate a unified patient-level risk estimate.

The current study focuses on ICU admission prediction as an indicator of severe COVID-19 progression and a clinically relevant surrogate endpoint associated with adverse outcomes. Although mortality represents an important final clinical outcome, direct multimodal risk prediction framework for severe COVID-19 outcomes requires separate outcome labeling and dedicated validation. Therefore, future work will extend the proposed multimodal framework toward joint prediction of ICU admission and mortality outcomes. In this work, ICU admission is considered as the primary prediction target because it represents a clinically significant marker of severe disease progression requiring intensive management.

To address this gap, this study proposes a weighted late fusion framework for ICU admission prediction using the CDSL (COVID Data for Shared Learning) dataset. A multimodal cohort of 784 patients containing both structured EHR and representative CT images was constructed for analysis. Three predictive settings were evaluated:

Practical and public health implications: beyond patient-level risk stratification, the proposed framework has potential implications for population-level public health monitoring. When clinical and imaging information can be aggregated at the city or district level in a privacy-preserving and interoperable manner, evidence generated from heterogeneous patient cohorts could support identification of areas experiencing increased healthcare burden and assist authorities in surveillance, resource allocation, and healthcare-capacity planning. Such data-driven approaches are also relevant to the development of resilient and digitally enabled urban health systems, where timely health indicators can contribute to evidence-based planning for future cities. However, these implications represent potential applications of the proposed framework rather than outcomes directly evaluated in the present patient-level study. Implementation at city or district scale would require appropriate data-sharing, interoperability, privacy, governance, and validation mechanisms.

## 2. Literature Review

Predicting severe COVID-19 progression and ICU admission has become an important research area in clinical decision support systems. Early studies mainly focused on structured electronic health records (EHRs), where demographic information, laboratory findings, and physiological measurements were used to estimate patient risk. Ruiz Giardin et al. [11] applied artificial intelligence models for mortality and ICU admission prediction in a large COVID-19 cohort. Daramola et al. [14] identified critical clinical variables linked with COVID-19 mortality risk. Similarly, Ghaffarzadeh-Esfahani et al. [13] showed that classical machine learning models remain effective for structured clinical prediction tasks using high-dimensional tabular data. Wichmann et al. [22] also demonstrated the value of federated learning for a COVID-19 multimodal risk prediction framework for severe COVID-19 outcomes across multiple hospitals.

Several studies have examined the clinical indicators associated with COVID-19 outcomes. Hsieh et al. [23] investigated the impact of comorbidities and clinical indicators on COVID-19 mortality. Dong et al. [24] showed that hematological markers can support an in-hospital multimodal risk prediction framework for severe COVID-19 outcomes in severe COVID-19 patients. Joseph et al. [25] used electronic health records to study health diagnoses associated with COVID-19 death in the United States. Chen et al. [26] further showed that the timing of clinical measurements can affect COVID-19 infection and multimodal risk prediction framework for severe COVID-19 outcomes, while Dehghani et al. [27] explored deep neural decision tree and forest models for predicting COVID-19 recovery or mortality.

Although EHR-based models provide useful prediction capability, they often fail to capture direct pulmonary severity, which is highly relevant in COVID-19 progression. Chest computed tomography (CT) provides detailed imaging evidence of lung infection, inflammation, and respiratory deterioration. De Smet et al. [12] showed that CT-derived biomarkers can support COVID-19 multimodal risk prediction framework for severe COVID-19 outcomes across multiple waves and regions. Ozawa et al. [15] further demonstrated the value of explainable AI techniques for COVID-19 severity prediction. Zhang et al. [16] also developed machine learning models for COVID-19 severity-risk prediction.

Several researchers investigated whether imaging and other non-tabular information can add value beyond structured clinical features. da Silva and Pazin-Filho [28] showed that unstructured clinical data processed through natural language processing can provide additional value in a COVID-19 multimodal risk prediction framework for severe COVID-19 outcomes. Lin et al. [17] showed that radiology reports and images can improve an ICU multimodal risk prediction framework for severe COVID-19 outcomes when combined with clinical information. Li et al. [18] demonstrated that longitudinal chest X-rays and EHR data can provide complementary information for predicting in-hospital outcomes in heart failure patients.

Recently, there has been a move towards multimodal prediction, where multiple forms of clinical data are used together. Wang and Li [19] integrated multimodal EHR data for a multimodal risk prediction framework for severe COVID-19 outcomes in ICU sepsis patients. Ruan et al. [20] proposed a multimodal ICU outcome prediction framework using structured EHR data and free-text notes. Rong et al. [29] developed a deep learning model for clinical outcome prediction using longitudinal inpatient EHR data. Engelke et al. [30] introduced FHIR-Former, showing how structured healthcare data and language-model-based approaches can support clinical prediction.

Fusion strategy selection is another open problem in multimodal healthcare prediction. In early fusion, raw features are first combined and then used for training, although this method is often associated with dimensional inconsistency and optimization problems. Late fusion strategies are often more stable because each modality can be optimized independently before predictions are combined [17,19]. Ruan et al. [20] also showed that multimodal frameworks can improve reliability by accounting for uncertainty when combining structured EHR data and free-text notes.

In recent years, additional research has also focused on multimodal ICU prediction and clinical outcome modelling. Wang et al. [21] introduced a causal relationship-guided deep learning framework for an ICU multimodal risk prediction framework for severe COVID-19 outcomes, suggesting that clinical knowledge can improve both performance and relevance in critical care prediction. Together, these studies show that multimodal and evidence-guided modelling can improve outcome prediction, but they also highlight the need for methods that remain interpretable enough for clinical use.

However, multiple research gaps still exist. The previous literature has mostly concentrated on a multimodal risk prediction framework for severe COVID-19 outcomes instead of ICU admission prediction, despite the fact that the latter constitutes a more clinically relevant task. Second, many existing studies have primarily used benchmark datasets or non-COVID multimodal settings rather than real-world COVID-19 multimodal datasets [17,18,19]. Finally, it is unclear whether multimodal fusion leads to significant improvement compared to a single powerful CT-based baseline with representative patient-level CT images. This study aims to fill those gaps by comparing EHR-only, CT-only, naïve fusion, and weighted late fusion models based on the CDSL multimodal COVID-19 dataset to examine whether multimodal fusion actually makes a difference for ICU admission prediction.

### Urban Public Health, Data Openness, and Practical Implications

The COVID-19 pandemic demonstrated that urban environments are particularly important contexts for understanding and managing infectious disease risks because of the concentration of population, economic activity, mobility, and healthcare demand within cities. Sharifi and Khavarian-Garmsir reviewed the early literature on COVID-19 and cities and identified four major thematic areas: environmental quality, socio-economic impacts, management and governance, and transportation and urban design. Their review further highlighted that the available evidence was disproportionately concentrated on environmental indicators, while issues related to urban governance, socio-economic vulnerability, transportation, and urban design remained comparatively less explored [31]. These observations emphasize the importance of integrating heterogeneous health and contextual information when developing evidence-based approaches for resilient urban public health management.

Data openness and integration represent another important component of this transition. Mak examined the COVID-19 pandemic across five selected East Asian cities and subsequently assessed the openness and integration of healthcare and environmental datasets. The report discusses the use of temporal and spatial information, pollution monitoring, numerical modelling, satellite remote sensing, and ground-based observations, while also identifying deficiencies in the availability and accessibility of pandemic-related healthcare and environmental data [32]. These observations highlight the importance of interoperable and sufficiently accessible data infrastructures for timely public health assessment and future city development.

In this context, the proposed multimodal clinical decision support framework can be viewed as a patient-level component within a broader evidence-driven public health ecosystem. When appropriately aggregated, anonymized, and governed, predictions derived from heterogeneous clinical and imaging cohorts could potentially contribute to higher-level surveillance and healthcare planning at city or district scales. Such an extension could support the identification of changing patterns of clinical risk, inform healthcare-capacity planning, and provide additional evidence for public health authorities during periods of increased disease burden. However, the present study does not directly evaluate city-level or district-level prediction, data openness, or urban planning outcomes. Therefore, these applications should be interpreted as potential future extensions rather than outcomes demonstrated by the current experiments. Future work should investigate interoperable, privacy-preserving mechanisms for connecting patient-level clinical decision support systems with broader urban health-data infrastructures.

## 3. Problem Statement and Proposed Methodology

### 3.1. Problem Statement

Early identification of COVID-19 patients who may require intensive care unit (ICU) admission is a major challenge in hospital resource planning and emergency clinical management. Failure to identify severe cases early can have important clinical consequences, including delays in ICU admission and ventilatory support, difficulties in treatment prioritization, and inefficient allocation of limited critical care resources. In rapidly deteriorating patients, such delays may also contribute to poorer clinical outcomes.

Predicting the need for ICU admission is therefore not simply a modeling task; it has direct implications for patient management and hospital resource planning [1]. Many conventional prediction approaches rely primarily on structured electronic health record (EHR) data, including patient demographics, vital signs, and laboratory findings [1]. These variables provide important information about a patient’s overall clinical and physiological condition, but they may not fully represent the extent of pulmonary involvement associated with COVID-19 [1]. Chest CT imaging provides a complementary source of information by capturing structural changes in the lungs, including patterns associated with inflammation and disease severity. However, imaging alone does not provide the broader physiological and biochemical information available in clinical records. This complementary relationship provides a strong rationale for combining EHR and CT data when predicting ICU admission [2,3].

The fundamental problem is this: the majority of existing research treats EHR data and CT imaging as two separate, unrelated problems, developing models that rely on just one data type instead of combining the strengths of both [1].

And in the cases where researchers have attempted multimodal fusion, the go-to method has usually been simple feature concatenation—which brings its own set of headaches, including dimensional mismatches, unstable training dynamics, and models that are difficult to interpret in practice [7,8]. What has not been clearly established yet is whether combining CT and clinical data actually beats a well-built CT-only model, particularly when working with realistic, patient-level CT imaging [1].

Therefore, the main research question of this study is as follows:

*Does multimodal fusion of structured EHR features and CT imaging improve a multimodal risk prediction framework for severe COVID-19 outcomes compared to unimodal models?*

Formally, given the patient-level dataset

$$D=\{X_{ehr},X_{ct},y\}$$

where

- $X_{ehr}$ represents structured emergency clinical features;
- $X_{ct}$ represents representative CT image features;
- *y* represents ICU admission outcome.

The objective is to verify whether

$$Performance\left(f_{fusion}\left(X_{ehr},X_{ct}\right)\right)>Performance\left(f_{ehr}\left(X_{ehr}\right)\right)$$

and

$$Performance\left(f_{fusion}\left(X_{ehr},X_{ct}\right)\right)>Performance\left(f_{img}\left(X_{ct}\right)\right)$$

for evaluation metrics such as precision, recall, F1-score, and ROC-AUC.

The ICU admission target is defined as follows:

$$y_{i}=\left\{\begin{array}{cc} 1, & \mathrm{if}\;\mathrm{ICU}\;\mathrm{admission}\;\mathrm{exists}\\ 0, & \mathrm{otherwise} \end{array}\right.$$

where $y_{i}=1$ indicates a severe patient requiring critical care support.

In practical terms, this formulation defines the evaluation procedure used in this study. For each patient *i*, the structured emergency clinical variables $X_{ehr,i}$ and the corresponding representative CT information $X_{ct,i}$ are paired with the observed ICU admission outcome $y_{i}$. Three prediction settings are then evaluated using the same patient-level evaluation cohort: an EHR-only model $f_{ehr}$, an imaging-only model $f_{img}$, and the proposed multimodal fusion model $f_{fusion}$. The outputs of these models are compared using ROC-AUC, precision, recall, and F1-score to determine whether integrating clinical and imaging information provides additional predictive value over either modality alone. Thus, the formulation provides the mathematical basis for the comparative experimental design rather than representing an additional prediction equation.

#### Data Preprocessing and Patient-Level Data Partitioning

A comprehensive preprocessing pipeline was applied before model development to ensure consistent integration of heterogeneous clinical and imaging information. Patient identifiers were used as the primary reference for associating structured electronic health record (EHR) information with corresponding computed tomography (CT) examinations. All records originating from the same patient were maintained within a single dataset partition to prevent information leakage between training and evaluation phases.

The complete dataset was divided into training, validation, and testing subsets using a patient-wise stratified splitting strategy. This approach ensured that patients with similar clinical episodes or multiple available records did not appear in more than one subset. Stratification was performed according to the target outcome distribution to maintain comparable class proportions across the different partitions.

For structured EHR variables, continuous clinical measurements were processed before model training. Missing numerical values were handled using median-based imputation derived exclusively from the training data. This prevented information from the validation or testing cohorts from influencing the preprocessing stage. Categorical variables were transformed into numerical representations before being provided to the prediction models.

The extracted clinical variables were subsequently organized into a structured feature representation containing demographic information and emergency department physiological measurements. This representation was used for developing the EHR-only prediction model.

### 3.2. Proposed Methodology

To address this problem, a multimodal weighted late fusion framework is proposed using the CDSL (COVID Data for Shared Learning) dataset. The methodology consists of three prediction stages: EHR-only prediction, CT-only prediction, and multimodal weighted late fusion.

#### 3.2.1. Methodology Overview

The proposed framework is formulated as a multimodal prediction function that integrates structured clinical information and CT-derived imaging representations. For each patient *i*, the input consists of two complementary modalities:

$$X_{i}=\left\{X_{ehr}^{i},X_{ct}^{i}\right\}$$

where $X_{ehr}^{i}$ represents the structured EHR feature vector and $X_{ct}^{i}$ represents the extracted CT image representation.

The EHR branch learns a mapping function:

$$f_{ehr}:X_{ehr}^{i}\to P_{ehr}^{i}$$

where $P_{ehr}^{i}$ denotes the probability prediction generated from clinical information.

Similarly, the imaging branch learns the following:

$$f_{ct}:X_{ct}^{i}\to P_{ct}^{i}$$

where $P_{ct}^{i}$ represents the probability derived from CT-based features.

The final multimodal prediction is obtained by combining both modality-specific outputs through weighted late fusion:

$$P_{fusion}^{i}=\alpha P_{ehr}^{i}+\left(1-\alpha \right)P_{ct}^{i}$$

where α controls the relative contribution of structured clinical and imaging evidence.

The final predicted outcome is determined using a classification threshold:

$$\hat{y_{i}}=\left\{\begin{array}{cc} 1, & P_{fusion}^{i}\geq 0.5\\ 0, & P_{fusion}^{i}<0.5 \end{array}\right.$$

This formulation enables independent evaluation of each modality and quantifies the contribution of multimodal evidence compared with unimodal prediction approaches.

The proposed workflow unfolds across three consecutive stages. In the first stage, structured EHR variables are processed to produce clinical risk estimates. The second stage converts CT images into feature representations using a pretrained ResNet50 feature extractor, which then feeds into a classification step. In the final stage, the probability outputs generated by both branches are combined via weighted late fusion, yielding the overall patient-level prediction.

Table 1 summarizes the overall predictive framework.

The ResNet-based imaging branch transforms the preprocessed CT images into learned feature representations that capture discriminative imaging patterns associated with ICU admission risk. These representations are subsequently incorporated into the multimodal prediction framework together with structured emergency clinical information. The purpose of the imaging branch is therefore not limited to direct image classification; it provides a learned representation of CT-derived information that can complement the physiological information contained in the clinical modality.

#### 3.2.2. EHR-Only Prediction

The first prediction branch of the proposed framework utilizes structured clinical information extracted from the electronic health records. The selected variables represent routinely collected emergency department measurements, including demographic characteristics and physiological indicators associated with patient deterioration.

The EHR feature set includes age, sex, blood pressure measurements, body temperature, heart rate, oxygen saturation (SpO_2_), and glucose levels. To capture the evolution of the patient’s clinical condition, both initial and final emergency department measurements were incorporated. The inclusion of these temporal endpoints allows the model to consider changes occurring during the early hospitalization period rather than relying only on admission characteristics.

The structured feature representation is defined as follows:

$$X_{ehr}=\left\{x_{1},x_{2},x_{3},\ldots ,x_{n}\right\}$$

where each $x_{i}$ represents an individual clinical variable.

A Gradient Boosting classifier was selected as the EHR-based prediction model due to its ability to capture nonlinear interactions among heterogeneous clinical variables. The model learns a mapping between the extracted EHR representation and the target clinical outcome:

$$f_{ehr}\left(X_{ehr}\right)\to y$$

where *y* represents the predicted outcome label.

The final EHR model performance was evaluated independently to quantify the predictive contribution of structured clinical information before integration with imaging-based features.

#### 3.2.3. CT-Only Prediction

The second prediction branch investigates the contribution of radiological information by utilizing chest CT images associated with individual patients. Since multiple CT images may exist for a single patient, a patient-level image selection strategy was adopted to reduce duplication effects and prevent biased evaluation.

Before feature extraction, CT images were subjected to standard preprocessing to ensure compatibility with the pretrained ResNet50 architecture. The selected CT slices were resized to the required input dimensions and intensity values were normalized before being provided to the feature extractor. These preprocessing steps reduce variability caused by differences in image scale and intensity distribution while maintaining the original structural information of the CT images.

Only CT examinations corresponding to the enrolled patients were considered. A representative CT image was selected from each patient’s available examination set, ensuring that every patient contributed a single imaging representation to the model. This strategy avoids over-representation of patients with larger numbers of available CT slices.

Before feature extraction, CT images were resized according to the input requirements of the convolutional neural network and normalized using the standard preprocessing procedure associated with the pretrained network.

For imaging-based prediction, one representative chest CT slice was selected for each patient to obtain a consistent patient-level imaging representation while avoiding over-representation of patients with multiple available slices. This approach reduces computational complexity and prevents potential patient-level bias during model evaluation.

Although the selected slice-based representation provides an efficient approach for evaluating the contribution of CT-derived features, it does not fully capture the three-dimensional distribution and progression of pulmonary abnormalities. Future work will investigate volumetric CT analysis approaches to incorporate complete spatial information from chest CT examinations.

A pretrained ResNet50 model was employed as a feature extractor by removing the original classification layer and utilizing the resulting convolutional representations. The use of pretrained weights provides a stable initialization for extracting general visual patterns from CT images while reducing the computational requirements associated with training a deep network from limited medical imaging data. The extracted image embedding is represented as follows:

$$X_{ct}=ResNet50\left(I_{i}\right)$$

where $I_{i}$ represents the selected CT image of patient *i*.

The extracted 2048-dimensional feature vector was subsequently provided to a Logistic Regression classifier:

$$f_{img}\left(X_{ct}\right)\to y$$

This branch evaluates the independent predictive capability of CT-derived imaging features without incorporating clinical information.

Although the pretrained ResNet50 architecture was not further fine-tuned using a COVID-19-specific CT cohort in this study, it provides a reproducible baseline for evaluating the contribution of CT-derived features. Future investigations will explore domain-specific transfer learning and fine-tuning strategies using larger annotated COVID-19 CT datasets to improve the representation of disease-specific pulmonary characteristics.

#### 3.2.4. Multimodal Weighted Late Fusion

To combine complementary information from clinical records and medical imaging, a weighted late fusion strategy was implemented. Instead of directly combining raw EHR variables with image embeddings, both modalities were first independently processed to generate outcome probabilities.

The probability estimated from the EHR branch is represented as follows:

$$P_{ehr}=f_{ehr}\left(X_{ehr}\right)$$

while the probability generated from the CT branch is defined as follows:

$$P_{img}=f_{img}\left(X_{ct}\right)$$

The final multimodal prediction probability is obtained through a weighted combination of both modality-specific predictions:

$$P_{final}=\alpha P_{ehr}+\left(1-\alpha \right)P_{img}$$

where α controls the relative contribution of structured clinical information and imaging information.

The fusion coefficient was optimized using the validation dataset by evaluating multiple candidate values and selecting the configuration providing the best balance between predictive discrimination and classification performance.

The final prediction was generated using the following decision threshold:

$$\hat{y}=\left\{\begin{array}{cc} 1, & P_{final}\geq 0.5\\ 0, & otherwise \end{array}\right.$$

This design enables direct comparison between individual modality performance and the integrated multimodal framework, allowing evaluation of whether combining clinical and radiological evidence improves prediction capability.

The weighted late fusion strategy provides a degree of modality-level interpretability by preserving the independent prediction contributions from EHR and CT branches before generating the final prediction. This enables analysis of the relative contribution of clinical and imaging evidence in the decision process.

#### 3.2.5. Model Training Configuration

All prediction models were trained using the same patient-level dataset partitions to ensure a fair comparison between unimodal and multimodal approaches. The training procedure was performed using only the training cohort, while the validation cohort was used for model selection and parameter optimization.

The Gradient Boosting model was trained using structured EHR variables, whereas the Logistic Regression classifier received the extracted CT feature embeddings generated by the ResNet50 feature extractor. Model parameters were optimized using validation performance to reduce overfitting and improve generalization.

For the multimodal model, different fusion weights were evaluated systematically. The optimal fusion coefficient was selected according to validation performance using evaluation measures including ROC-AUC and F1-score.

The final trained models were evaluated on the independent testing cohort, which remained unseen during training and optimization. This evaluation strategy provides an unbiased assessment of the proposed multimodal framework. The experiments were implemented using a consistent preprocessing and training pipeline for both clinical and CT modalities. Clinical variables were preprocessed prior to model training, while CT images underwent standardized image preprocessing before being provided to the imaging model. Model development, multimodal fusion, and hyperparameter selection were performed using the training/validation data, while the held-out test cohort was reserved exclusively for final evaluation. All data partitioning was performed at the patient level to prevent information leakage between cohorts.

## 4. Dataset Description

This study uses the CDSL (COVID Data for Shared Learning) dataset, a large-scale multimodal COVID-19 clinical dataset developed from HM Hospitales, Spain. The dataset contains structured electronic health records (EHRs), diagnostic reports, laboratory findings, medication history, vital signs, and extensive medical imaging information for COVID-19 patients. It provides a suitable foundation for multimodal clinical decision support research because it combines both numerical hospital records and radiological imaging at the patient level.

The original dataset contains information for 4479 patients and 1,444,764 DICOM imaging records. Since the objective of this study is multimodal risk prediction framework for severe COVID-19 outcomes using CT imaging and structured clinical variables, only patients with available chest CT imaging and complete emergency clinical features were considered for the multimodal cohort construction.

To ensure consistent multimodal representation learning, this study included only patients with both structured EHR information and corresponding CT imaging data available. This selection allowed direct comparison between unimodal and multimodal approaches under a complete-data setting. However, this criterion may reduce applicability in emergency scenarios where one or more clinical modalities may be unavailable.

Missing values within the available structured EHR variables were handled during preprocessing using an appropriate imputation strategy before model training. The same preprocessing procedure was applied consistently across training and testing cohorts.

### 4.1. Structured Clinical Records

The structured patient-level information was obtained from the file patient_01.csv, which contains 34 clinical attributes including demographic information, admission details, ICU records, discharge outcomes, emergency department vital signs, and mechanical ventilation status.

The major clinical variables include:

- Age;
- Sex;
- ICU admission date;
- ICU stay duration;
- Discharge destination;
- Blood pressure (maximum and minimum);
- Body temperature;
- Heart rate;
- Oxygen saturation (SpO_2_);
- Blood glucose.

Both first and last emergency measurements were included to preserve disease progression information before ICU transfer.

### 4.2. Medical Imaging Records

Imaging metadata was obtained from CDSL-1.0.0-dicom-metadata.csv, which contains 15 imaging-related attributes for 1,444,764 medical imaging studies (Table 2). Important metadata fields include:

- Patient ID;
- Study ID;
- Image ID;
- Modality;
- Study Description;
- Body Part;
- View Position;
- Patient Position.

The modality distribution analysis showed that CT imaging dominates the dataset, making it highly suitable for a CT-based multimodal modeling.

Since CT represented the dominant imaging modality, this study focused exclusively on chest CT-based multimodal prediction rather than chest X-ray classification.

### 4.3. Outcome Label Construction

The target variable for this study is a multimodal risk prediction framework for severe COVID-19 outcomes. ICU admission was defined using the availability of the ICU admission date field:

$$y_{i}=\left\{\begin{array}{cc} 1, & \mathrm{if}\;\mathrm{ICU}\;\mathrm{admission}\;\mathrm{exists}\\ 0, & \mathrm{otherwise} \end{array}\right.$$

where $y_{i}=1$ represents patients requiring intensive care support.

Mortality was also constructed as a secondary outcome using discharge destination:

$$y_{mortality}=\left\{\begin{array}{cc} 1, & \mathrm{if}\;\mathrm{discharge}\;\mathrm{status}\;=\;\mathrm{Death}\\ 0, & \mathrm{otherwise} \end{array}\right.$$

However, ICU admission was selected as the primary prediction target because it is more clinically actionable for hospital resource planning.

### 4.4. Multimodal Cohort Construction

From the complete dataset of 4479 patients, only patients with both representative CT images and complete structured EHR records were selected. One representative CT image per patient was extracted to avoid duplication bias and patient-level leakage during training.

After multimodal filtering, the final study cohort consisted of

n=784

patients.

The final class distribution for ICU prediction is shown in Table 3.

Similarly, mortality distribution was as follows:

- Alive/Non-death: 695;
- Death: 89.

This multimodal cohort forms the final dataset:

$$D_{multimodal}=\left\{X_{ehr},X_{ct},y_{ICU}\right\}$$

where $X_{ehr}$ represents structured clinical features, $X_{ct}$ represents representative CT image embeddings, and $y_{ICU}$ represents ICU admission outcome.

Because this dataset offers a balanced combination of structured EHR and CT imaging data, it serves as a strong testbed for determining whether multimodal fusion delivers meaningful gains over models that rely on a single data type alone.

The framework proposed in this study was built and tested using the CDSL dataset, which offers a clearly defined patient cohort suited to examining multimodal prediction of ICU admission in COVID-19 cases. That said, since evaluation was confined to this one dataset, the results obtained should be understood as specific to the characteristics of this particular population, rather than assumed to generalize broadly.

#### Patient-Level Dataset Partitioning and Leakage Prevention

To ensure reliable evaluation and prevent information leakage, all dataset partitioning procedures were performed at the patient level rather than at the individual record or image level (Table 4). Since multiple clinical observations and CT images may be associated with a single patient, splitting data at the image level could result in information from the same patient appearing in both training and testing subsets.

Therefore, each patient was assigned exclusively to one dataset partition, ensuring that all corresponding EHR records and CT images remained within the same subset. The dataset was divided into training, validation, and testing cohorts while preserving the distribution of the target outcome across all partitions.

The preprocessing pipeline was applied after dataset partitioning. Statistical parameters used for feature normalization and missing-value handling were estimated only from the training cohort and subsequently applied to validation and testing samples. This approach prevents indirect information transfer from evaluation data into the model development process.

## 5. Experimental Setup and Results

### 5.1. Experimental Setup

The main objective of this study is to evaluate whether multimodal fusion of structured electronic health records (EHRs) and chest CT imaging improves a multimodal risk prediction framework for severe COVID-19 outcomes compared to unimodal prediction models. Three predictive settings were evaluated:

$$f_{ehr}\left(X_{ehr}\right),$$

$$f_{img}\left(X_{ct}\right),$$

and

$$f_{fusion}\left(X_{ehr},X_{ct}\right)$$

where $X_{ehr}$ represents structured emergency clinical features and $X_{ct}$ represents representative CT image features.

The final multimodal cohort consisted of

n=784

patients with both structured EHR records and representative CT images.

The ICU admission target variable was defined as follows:

$$y_{i}=\left\{\begin{array}{cc} 1, & \mathrm{if}\;\mathrm{ICU}\;\mathrm{admission}\;\mathrm{exists}\\ 0, & \mathrm{otherwise} \end{array}\right.$$

with the following class distribution:

649Non-ICUpatients,135ICUpatients

**Figure 1:**
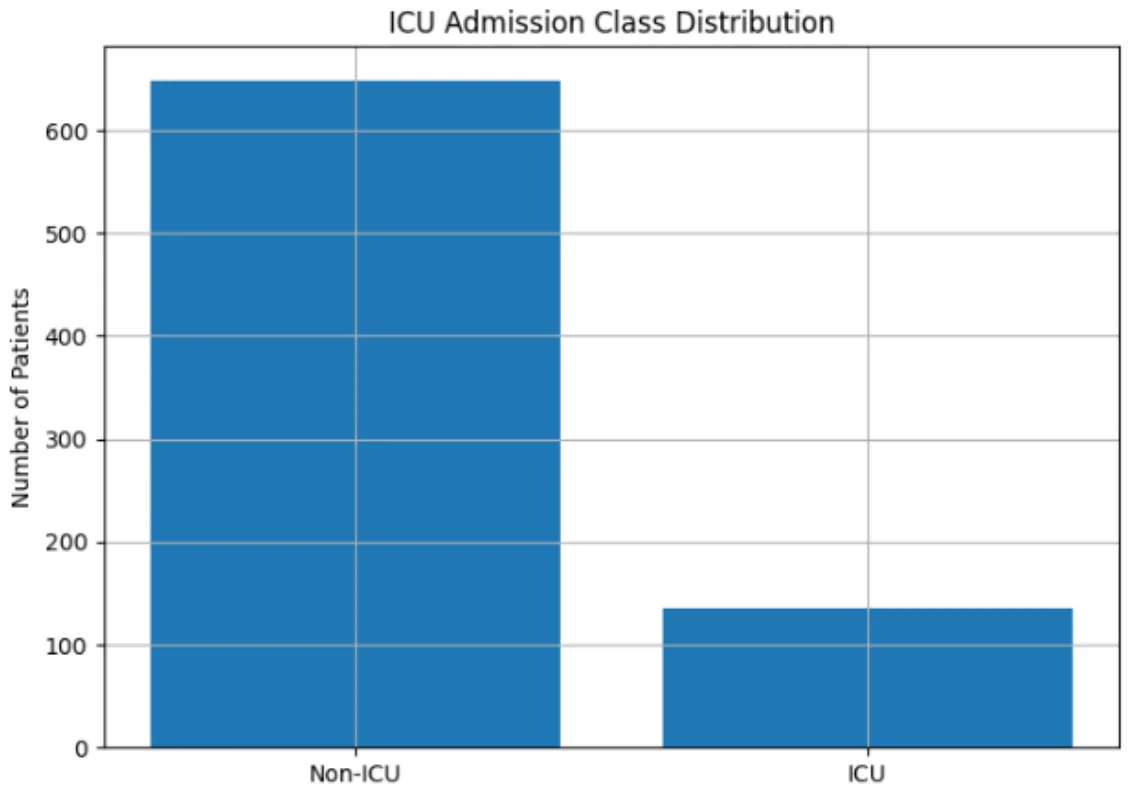
ICU admission class distribution.

Figure 1 shows the class imbalance of the final ICU prediction cohort. The moderate imbalance justifies the use of F1-score and recall as important evaluation metrics in addition to accuracy.

The dataset was divided using a stratified train–test split with

80%Training,20%Testing

to preserve class balance during evaluation.

### 5.2. Model Configuration

#### 5.2.1. EHR-Only Model

Structured clinical features including age, sex, blood pressure, temperature, heart rate, oxygen saturation, and glucose levels were used as input. Both first and last emergency measurements were included.

A Gradient Boosting Classifier was trained for

$$f_{ehr}\left(X_{ehr}\right)\to y$$

because of its strong performance on nonlinear structured healthcare data.

#### 5.2.2. CT-Only Model

One representative CT image per patient was selected. Pretrained ResNet50 was used as a deep feature extractor by removing the final classification layer and generating a 2048-dimensional embedding:

$$X_{ct}=ResNet50\left(I_{i}\right)$$

where $I_{i}$ denotes the representative CT image of patient *i*.

A Logistic Regression classifier was then trained for

$$f_{img}\left(X_{ct}\right)\to y$$

to evaluate independent CT predictive strength.

The clinical interpretation of the incremental benefit of multimodal integration warrants consideration. The CT-only model achieved an ROC-AUC of 0.772, while the multimodal model achieved 0.782, representing an absolute improvement of 0.010. This relatively modest gain should be interpreted in the context of the strong predictive contribution of CT imaging observed in this study. Because CT already captures substantial information associated with the evaluated outcome, the additional contribution of structured clinical variables may be incremental rather than large. Nevertheless, clinical variables provide complementary physiological and contextual information that is not directly represented in CT images. The observed improvement therefore suggests potential complementary value from multimodal integration rather than a substantial replacement of CT-based assessment. Importantly, an improvement in ROC-AUC alone does not establish clinical utility or clinical superiority. Determining whether the incremental predictive information provided by multimodal integration leads to meaningful changes in clinical decision-making would require evaluation using clinically relevant operating thresholds, calibration and decision-analytic measures, as well as prospective assessment in independent clinical populations.

#### 5.2.3. Weighted Late Fusion

Instead of direct feature concatenation, weighted late fusion was applied using probability-level integration

$$P_{final}=\alpha P_{ehr}+\left(1-\alpha \right)P_{img}$$

where:

- $P_{ehr}$ = probability from EHR model;
- $P_{img}$ = probability from CT model;
- α = modality contribution weight.

The optimal value of α was determined experimentally by maximizing F1-score.

The best value was found as follows:

α=0.50

which indicates equal contribution from both modalities.

**Figure 2:**
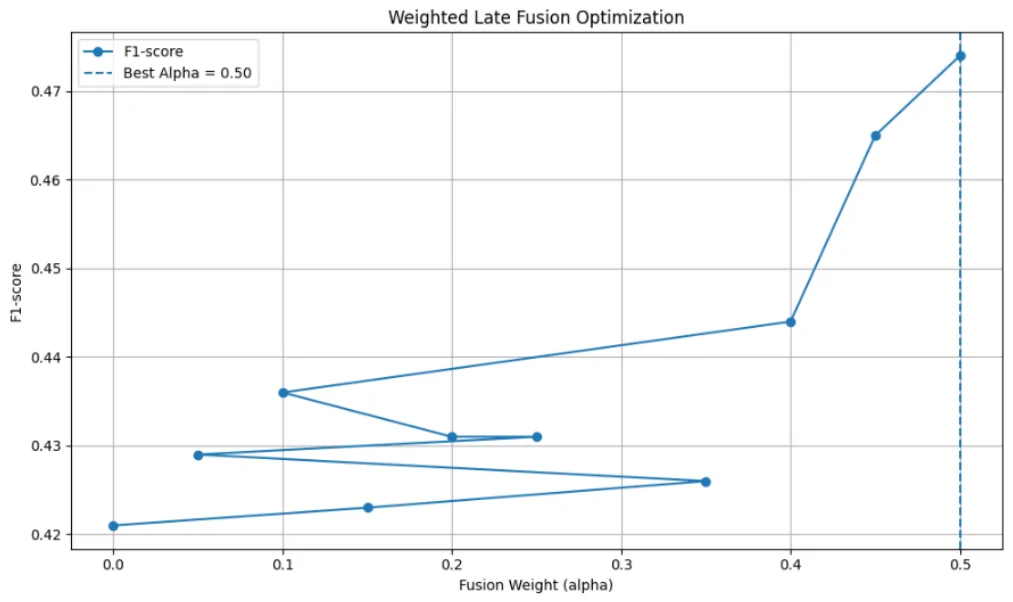
Weighted late fusion optimization.

Figure 2 illustrates the optimization of fusion weight α, where the best F1-score was achieved at α=0.50, indicating balanced contribution from EHR and CT modalities.

The CT-only model achieved an ROC-AUC of 0.772, whereas the multimodal model achieved an ROC-AUC of 0.782, corresponding to an absolute improvement of 0.010. In comparison, the multimodal model demonstrated an absolute ROC-AUC improvement of 0.067 over the EHR-only model (0.715).

### 5.3. Evaluation Metrics

Performance was evaluated using:

- Precision;
- Recall;
- F1-score;
- ROC-AUC.

Special emphasis was placed on ICU-positive recall and F1-score because false negative ICU cases are clinically more dangerous than false positives.

### 5.4. Results and Discussion

#### 5.4.1. EHR-Only Baseline

The EHR-only model produced the following confusion matrix:

$$\left[\begin{array}{cc} 123 & 7\\ 21 & 6 \end{array}\right]$$

The model achieved:

- Precision = 0.46;
- Recall = 0.22;
- F1-score = 0.30;
- ROC-AUC = 0.715.

The results indicate strong specificity but poor ICU-positive sensitivity, showing that structured EHR alone is insufficient for reliable ICU prediction.

#### 5.4.2. CT-Only Baseline

The CT-only model produced the following confusion matrix:

$$\left[\begin{array}{cc} 112 & 18\\ 15 & 12 \end{array}\right]$$

The model achieved:

- Precision = 0.40;
- Recall = 0.44;
- F1-score = 0.42;
- ROC-AUC = 0.772.

CT imaging performed better than the EHR-only baseline, suggesting that imaging features related to pulmonary severity provide a stronger signal for predicting ICU admission than the clinical variables used in this study.

**Figure 3:**
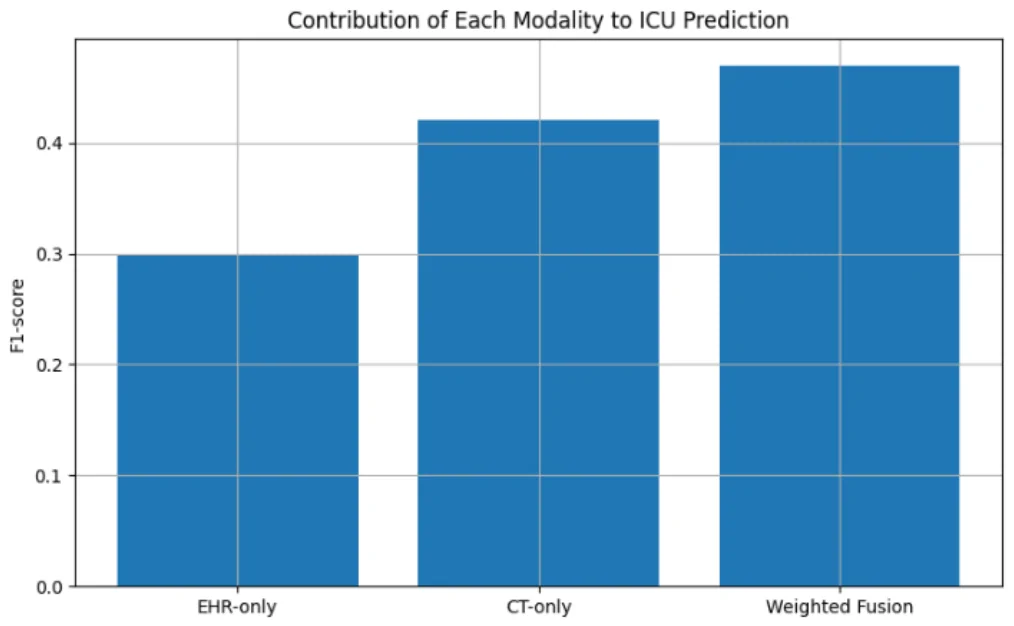
Contribution of each modality to ICU prediction.

Figure 3 shows the contribution of each modality to the prediction task. Among the evaluated approaches, the weighted fusion model achieved the highest overall F1-score, indicating that combining information from both modalities provided better predictive performance than using either modality alone.

#### 5.4.3. Naive Feature Fusion

Direct feature concatenation of EHR and CT embeddings produced

$$\left[\begin{array}{cc} 129 & 1\\ 20 & 7 \end{array}\right]$$

with:

- Precision = 0.88;
- Recall = 0.26;
- F1-score = 0.40;
- ROC-AUC = 0.772.

Although precision improved, recall remained weak, indicating that naive fusion does not sufficiently improve multimodal prediction.

#### 5.4.4. Weighted Late Fusion

The proposed weighted late fusion model produced the best overall performance with the confusion matrix:

$$\left[\begin{array}{cc} 128 & 2\\ 18 & 9 \end{array}\right]$$

The final results were:

- Precision = 0.82;
- Recall = 0.33;
- F1-score = 0.47;
- ROC-AUC = 0.782.

The model improved the ICU-positive F1-score as follows

0.42→0.47

and improved ROC-AUC as follows

0.772→0.782

compared to the CT-only baseline.

**Figure 4:**
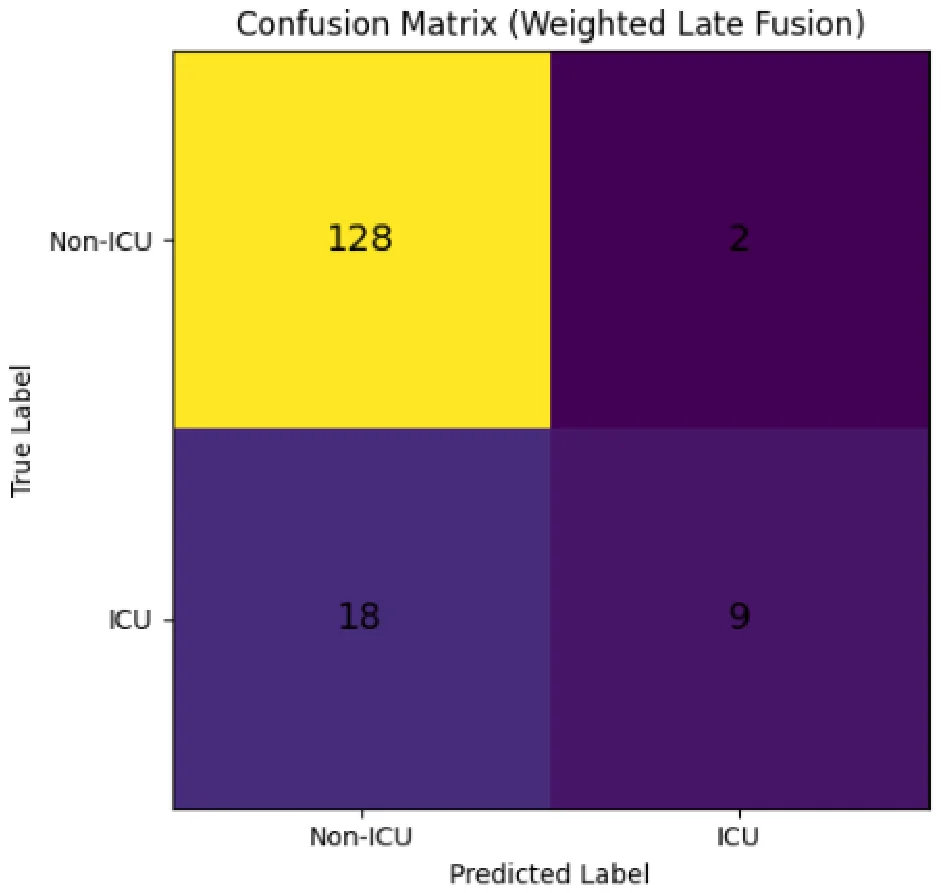
Confusion matrix of weighted late fusion model.

Figure 4 presents the confusion matrix of the best weighted fusion model, showing improved ICU-positive detection while maintaining high specificity.

### 5.5. Comparative Performance Analysis

Table 5 summarizes the final model comparison.

**Figure 5:**
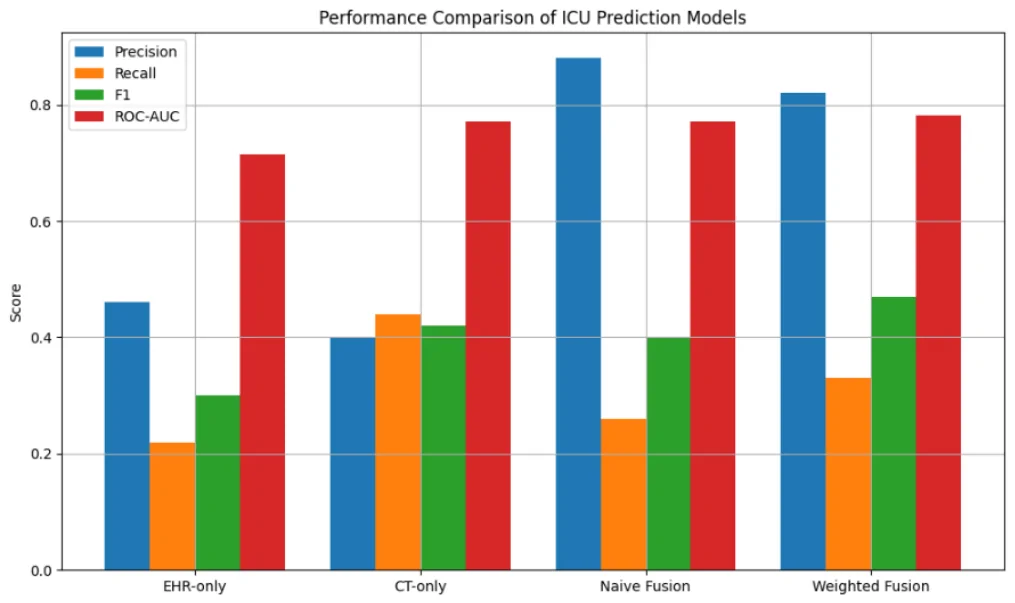
Performance comparison of ICU prediction models.

Figure 5 provides a visual comparison of precision, recall, F1-score, and ROC-AUC across all predictive models.

The results confirm

$$Performance\left(f_{fusion}\right)>Performance\left(f_{ehr}\right)$$

and

$$Performance\left(f_{fusion}\right)>Performance\left(f_{img}\right)$$

particularly for F1-score and ROC-AUC.

This demonstrates that weighted late fusion provides clinically meaningful improvement over unimodal baselines while maintaining model interpretability and deployment feasibility. The improvement over CT-only prediction was modest (ΔAUROC = 0.010), whereas the improvement over EHR-only prediction was larger (ΔAUROC = 0.067). This suggests that CT imaging already captures a substantial proportion of the predictive signal, while structured clinical variables may provide additional complementary information. However, because the present evaluation reports point estimates from a single held-out test cohort, the magnitude of these differences should not be interpreted as evidence of statistical superiority without formal uncertainty estimation.

## 6. Limitations and Future Directions

The findings of this study show that combining clinical information with CT imaging can be useful for COVID-19 outcome prediction. However, there are several limitations that need to be considered when interpreting the results.

One of the main limitations is that all experiments were carried out on a single dataset. This makes it difficult to know whether the same performance would be achieved in patients treated at other hospitals or in different populations. Patient characteristics, disease prevalence, treatment practices, and even routine clinical workflows can differ considerably between institutions. These differences may affect how a model behaves once it is taken outside the environment represented in the training data. Validation on independent cohorts from multiple hospitals is therefore an important next step. Such testing would provide a much clearer picture of how well the framework generalizes before it is considered for clinical use.

CT imaging introduces another source of uncertainty. Images collected at different hospitals are rarely acquired under exactly the same conditions. Scanner type, acquisition protocol, reconstruction settings, and image quality can all vary, and these differences may influence the features learned from the scans. We used a common preprocessing procedure to reduce some of this variation, but preprocessing alone cannot guarantee that scanner- or site-related differences have been removed. Testing the model on CT scans acquired using different scanners and imaging protocols would help determine how sensitive the imaging component is to these changes.

There are also limitations in the way the two modalities are represented and combined. In the current framework, EHR and CT information are brought together at the patient level. This provides a practical way of combining the two sources, but it does not capture every type of information that may be relevant to disease progression. For example, changes in clinical measurements over time, treatment or medication response, genomic information, and information contained in free-text clinical notes are not represented in the present model. Including some of these sources in future studies may provide a more complete description of a patient’s condition.

The CT pipeline could also be developed further. The current analysis is based on selected CT slices rather than the complete three-dimensional scan. Although these slices contain useful pulmonary information, some abnormalities may appear outside the selected regions, and relationships between findings across slices are not fully represented. Using the complete CT volume would allow future models to learn three-dimensional patterns throughout the lungs. Lung segmentation, artifact correction, and more targeted noise-reduction methods could also be investigated to determine whether they improve the quality of the imaging representations.

Weighted late fusion was useful in this study because the predictions from the EHR and CT models remained separate before being combined. This makes it possible to see how much each modality contributes to the final prediction. At the same time, this form of interpretability is fairly broad. It does not tell a clinician which laboratory measurement or clinical variable influenced a prediction, nor does it show which part of a CT image contributed most strongly to the imaging model’s decision. A useful extension would therefore be to add feature-level and image-level explanations. SHAP, for example, could be used to examine the contribution of individual EHR variables, while methods such as Grad-CAM could highlight image regions that have a strong influence on CT-based predictions. These explanations would also need to be assessed carefully to determine whether they are clinically meaningful rather than simply visually convincing.

The fusion mechanism itself leaves room for further investigation. The current approach assigns weights when combining the predictions of the two modalities, but the same weighting strategy is applied across patients. In practice, the usefulness of each source may differ from case to case. For one patient, CT findings may provide the strongest evidence, whereas laboratory measurements or vital signs may be more informative for another. Patient-specific fusion could account for this difference by adjusting the contribution of each modality according to the available evidence. More complex approaches, including cross-attention, multimodal transformers, and joint representation learning, could also be compared with late fusion. However, any improvement in predictive performance would need to be considered alongside the added computational cost and loss of interpretability that may come with more complex architectures.

The reported results also require stronger statistical validation. Multiple evaluation metrics were used in the present experiments, but a single performance estimate does not show how much the results might change under a different division of the data. Repeating the experiments across several train–test splits and reporting confidence intervals would provide a better indication of performance stability. This would help establish whether the observed improvements are consistent or are partly dependent on a particular data partition.

Finally, the framework should be viewed as a decision support tool rather than an alternative to clinical judgment. Performance on a retrospective dataset does not by itself demonstrate that a model will be useful in routine care. Prospective evaluation with patients encountered in real clinical practice would be required to understand how the system behaves under real-world conditions. Practical issues are equally important. The framework would need to work with existing hospital information systems, fit within established clinical workflows, and provide its predictions in a form that clinicians can interpret and use without creating unnecessary workload. These questions fall outside the scope of the present study but are essential if the framework is eventually to move from experimental evaluation to clinical application. Although the present study demonstrates the predictive contribution of the ResNet-based imaging branch through quantitative model evaluation, explicit spatial attribution and temporal visualization were not part of the original experimental protocol. Future work will therefore incorporate techniques such as class-activation mapping and longitudinal visualization to provide more detailed interpretation of image regions and temporal clinical patterns contributing to risk predictions.

## 7. Conclusions and Future Work

This study examined whether clinical records and CT imaging can provide more useful information for COVID-19 outcome prediction when they are considered together rather than separately. To investigate this, we compared models trained on structured EHR data, CT images, and a combination of the two. Across these experiments, the multimodal approach produced the strongest overall results, with weighted late fusion achieving the best performance among the evaluated strategies.

An important observation from these results is that the two modalities appear to contribute different information to the prediction task. Clinical records describe the patient’s broader physiological condition through variables such as laboratory measurements and other structured clinical information, while CT images provide a direct view of pulmonary involvement. Neither source gives a complete description of the patient on its own. Bringing them together allowed the framework to use information that would otherwise remain separated.

Weighted late fusion also provided a practical way of carrying out this integration. Instead of forcing the EHR and imaging information into a single representation at an early stage, each modality was modeled independently and their predictions were combined afterward. This retained the contribution of the individual modalities while still benefiting from the information available in both. The results obtained in this study support this approach for the dataset examined, although they should not yet be interpreted as evidence that the same performance will necessarily be achieved in other clinical settings.

There are several ways in which the work can now be extended. The most immediate requirement is external validation using data from different hospitals and patient populations. Moving from selected CT slices to complete volumetric scans would also allow the imaging model to make use of spatial information that is not represented in the current pipeline. Similarly, EHR modeling could be extended beyond static patient measurements to include changes over time, which may be particularly useful when a patient’s condition is evolving.

The way information is fused could also become more patient-specific. Rather than using the same weighting strategy for every case, future models could learn when greater emphasis should be placed on clinical information and when imaging findings should have a larger influence. At the same time, more detailed explanation methods are needed so that clinicians can examine not only the contribution of each modality but also the individual clinical variables and CT regions behind a prediction.

Future studies could also move beyond the current prediction task. The same multimodal setting could be investigated for outcomes such as mortality, clinical deterioration, or other indicators of disease progression. Ultimately, however, the value of such a system will depend on more than predictive performance. Prospective testing, external validation, and evaluation within actual hospital workflows will be needed to determine whether the framework can provide useful information to clinicians at the point of care. The present study provides an initial step in that direction by showing that clinical and radiological information can complement one another when modeled jointly for patient-level COVID-19 outcome prediction. Future studies should therefore evaluate the proposed framework using independent external cohorts and prospective clinical validation. Additional assessment of calibration, decision-curve-based net benefit, clinically relevant risk thresholds, and clinician-in-the-loop evaluation would help determine whether the observed incremental predictive performance translates into meaningful improvements in clinical decision-making.

## Figures and Tables

**Table 1:** Overview of the proposed methodology.

Stage	Input	Model/Method
Stage 1	Structured EHR Features	Gradient Boosting
Stage 2	Representative CT Images	ResNet50 + Logistic Regression
Stage 3	EHR + CT Probabilities	Weighted Late Fusion

**Table 2:** Imaging modality distribution.

Modality	Count
CT	1,440,156
CR	3197
DX	1411

**Table 3:** Final ICU admission class distribution.

Class	Count
Non-ICU Patients	649
ICU Patients	135
Total	784

**Table 4:** Patient-level dataset partitioning.

Subset	Allocation	Purpose
Training	80%	Model training and parameter optimization
Testing	20%	Final performance evaluation on unseen patients

**Table 5:** Performance comparison of ICU prediction models.

Model	Precision	Recall	F1	ROC-AUC
EHR-only	0.46	0.22	0.30	0.715
CT-only	0.40	0.44	0.42	0.772
Naive Fusion	0.88	0.26	0.40	0.772
Weighted Fusion	**0.82**	0.33	**0.47**	**0.782**

## Data Availability

Dataset available on request from the authors.
